# Laser Ablation of Silicon Nanoparticles and Their Use in Charge-Coupled Devices for UV Light Sensing via Wavelength-Shifting Properties

**DOI:** 10.3390/nano13222915

**Published:** 2023-11-08

**Authors:** Algirdas Lazauskas, Dovilė Gimžauskaitė, Mindaugas Ilickas, Liutauras Marcinauskas, Mindaugas Aikas, Brigita Abakevičienė, Dmytro Volyniuk

**Affiliations:** 1 Institute of Materials Science, Kaunas University of Technology, K. Baršausko 59, LT51423 Kaunas, Lithuania; mindaugas.ilickas@ktu.edu (M.I.); brigita.abakeviciene@ktu.lt (B.A.); 2 Plasma Processing Laboratory, Lithuanian Energy Institute, Breslaujos 3, LT44403 Kaunas, Lithuania; dovile.gimzauskaite@lei.lt (D.G.); liutauras.marcinauskas@lei.lt (L.M.); mindaugas.aikas@lei.lt (M.A.); 3 Department of Polymer Chemistry and Technology, Kaunas University of Technology, K. Baršausko 59, LT51423 Kaunas, Lithuania; dmytro.volyniuk@ktu.lt

**Keywords:** silicon, nanoparticles, laser, ablation, luminescence, thin film, UV, sensing, wavelength-shifting

## Abstract

This study explores the controlled laser ablation and corresponding properties of silicon nanoparticles (Si NP) with potential applications in ultraviolet (UV) light sensing. The size distribution of Si NPs was manipulated by adjusting the laser scanning speed during laser ablation of a silicon target in a styrene solution. Characterization techniques, including transmission electron microscopy, Raman spectroscopy, and photoluminescence analysis, were employed to investigate the Si NP structural and photophysical properties. Si NP produced at a laser scanning speed of 3000 mm/s exhibited an average diameter of ~4 nm, polydispersity index of 0.811, and a hypsochromic shift in the Raman spectrum peak position. Under photoexcitation at 365 nm, these Si NPs emitted apparent white light, demonstrating their potential for optoelectronic applications. Photoluminescence analysis revealed biexponential decay behavior, suggesting multiple radiative recombination pathways within the nanoscale structure. Furthermore, a thin film containing Si NP was utilized as a passive filter for a 2nd generation CCD detector, expanding the functionality of the non-UV-sensitive detectors in optics, spectrometry, and sensor technologies.

## 1. Introduction

In the ever-evolving landscape of particle physics and scientific exploration, the detection of ultraviolet (UV) photons has emerged as a pivotal component in a multitude of experiments and applications. From unraveling the mysteries of Cherenkov radiation [1,2,3] in crystals to shedding light on enigmatic dark matter [4,5,6] and studying elementary particle decay [7,8], UV photon detection has become indispensable. Beyond the boundaries of particle physics, the utility of UV photon detection extends to a wide array of civil and military applications. From chemical and biological research [9,10,11,12], where the absorption of UV light plays a critical role, to fire detection systems [13,14,15] that safeguard lives and property, the need for precise UV photon detection is undeniable. Furthermore, UV light plays a vital role in monitoring plasma processes [16,17,18], advancing optical communication technologies [19,20,21], and calibrating light sources [22,23,24], making it a linchpin in various scientific and industrial domains.

UV light detection is traditionally performed using photomultipliers [25,26], thermometric detectors [27], narrow-bandgap semiconductor photodiodes [28,29], or charge-coupled device (CCD) detectors [30]. Photomultipliers provide high amplification and low signal noise but are fragile and space-consuming devices requiring high-power sources. Thermometric detectors (i.e., pyrometers and bolometers) are often used in the UV range as calibration standards. Although they are useful as radiometric standards, these detectors are very slow, and their response is not wavelength-dependent. Semiconductor photon detectors are small, lightweight, fast (with high speed), and insensitive to magnetic fields. However, UV photon detectors using semiconductor materials have certain limitations associated with silicon technology. For high-sensitivity applications, the active region of the photon detector must be cooled to reduce dark current [31,32], as the detector behaves as a “trap for impurities”, resulting in a gradual reduction in sensitivity. New UV light sensing materials capable of directly detecting UV photons and/or absorbing them by emitting electromagnetic waves in the visible light spectrum are needed. These emitted waves can be measured using current photon detectors, eliminating the necessity for an active region cooling system.

This article delves into an innovative approach that seeks new ways of UV light sensing by harnessing the unique properties of silicon nanoparticles. Silicon nanoparticles exhibit a unique sensitivity to UV light due to a phenomenon known as the “quantum confinement effect” [33,34]. This effect arises when the dimensions of a material are reduced to nanoscale dimensions, typically in the range of a few nanometers or smaller [35]. In bulk silicon, the electronic bandgap—the energy difference between the valence band and the conduction band—determines its optical properties. However, as silicon nanoparticles shrink in size, the energy levels of their electrons become quantized, leading to a substantial increase in the bandgap energy [36,37]. This elevated bandgap means that silicon nanoparticles can absorb higher-energy photons, including those in the UV range. Quantum confinement further influences the emission properties of silicon nanoparticles. When excited by UV light, silicon nanoparticles re-emit energy as visible light, the wavelength of which depends on the size of the nanoparticles [38]. 

Various methods exist for synthesizing silicon nanoparticles, encompassing chemical, physical, and physicochemical approaches. Chemical methods involve reducing silicon halides or metal silicides [33,39], while physical methods include laser ablation [40,41], ball milling [42,43], and thermal treatment [44,45]. Additionally, physicochemical techniques include electrochemical [46], solvothermal synthesis [47], and sol–gel methods [48]. Among these methods, laser ablation stands out as an exceptionally environmentally friendly and sustainable approach. It is used not only for nanoparticle synthesis; laser ablation applications encompass a broad range of uses, from precise material processing and analytical chemistry techniques to medical procedures and even material recrystallization [49]. In laser ablation, nanoparticles are generated by ablating a target submerged in a liquid using a high-power laser. Notably, the resulting nanoparticles exhibit distinctive properties that set them apart from those produced through chemical routes. Their shapes are influenced by the solvent used during ablation, and they are formed without agglomeration, an advantage not replicated by chemical processes. Moreover, this method is straightforward, requiring no surface activation materials or chemical additives for nanoparticle colloid extraction.

The key to achieving desired nanoparticle properties lies in effectively controlling the laser ablation process, which hinges on factors such as ablation duration, speed, pulse frequency, wavelength, and power density. These parameters dictate variations in the size, morphology, degree of crystallinity, and other physical and chemical attributes of the silicon nanoparticles [50,51,52]. 

Herein, pulsed laser ablation was employed to produce silicon nanoparticles (Si NP) in a styrene solution. The size distribution of Si NPs was modulated by adjusting the laser scanning speed during synthesis. Transmission electron microscopy (TEM), Raman spectroscopy, and photoluminescence analysis were used for Si NP characterization. Under photoexcitation at 365 nm, Si NP emitted apparent white light, and photoluminescence analysis revealed biexponential decay behavior. Free-standing thin films containing Si NP were fabricated using cyclo olefin polymer as a matrix. Their photophysical were found to be slightly different as compared to Si NP colloidal dispersions. Furthermore, our research extends beyond fundamental characterization to practical applications. We demonstrate the utility of Si NP by using them as passive filters for 2nd generation CCD detectors. The innovative configuration allows non-sensitive CCD detectors to detect UV light through the wavelength-shifting properties of Si NPs. This practical application exemplifies the transformative potential of Si NP in enhancing the functionality of existing technologies and could find applications in fields ranging from optics to spectrometry and sensor technologies.

## 2. Materials and Methods

Monocrystalline Si(1 0 0) wafers (Microchemicals GmbH, Ulm, Germany), with a thickness = 525 ± 25 μm, p-type (Boron) were used as a target material in laser ablation experiments. Styrene of analytical grade (CAS No. 100-42-5, ReagentPlus^®^, containing 4-tert-butylcatechol as a stabilizer, ≥99%, Sigma-Aldrich, Saint Louis, MO, USA) was used as a solvent for dispersing silicon nanoparticles (Si NP) during laser ablation. Cyclo olefin polymer ZEONEX^®^ (ZEON Corp., Tokyo, Japan) of grade 480R was used for the formation of thin films containing Si NP. 

Silicon nanoparticles were produced using pulsed laser ablation. In each trial, a pristine silicon wafer (polished side facing the incident laser beam) measuring 12 mm × 12 mm was placed at the bottom of a 10 mL glass bottle filled with 4 mL styrene solution. The bottle was then placed in the glass jar containing a small amount of water, which acted as a cooling agent for the styrene solution. Laser ablation was performed using an Nd:YAG fiber laser operating at 20 W power with a wavelength of 1064 nm (Shandong Reaying Machinery Co., Ltd., Jinan, China). The laser had a pulse duration (full width at half maximum) of 1.0 μs, operated at a repetition rate of 40 kHz, and utilized a 17.5 cm focal length. The laser spot size was ~20 μm. The laser beam was programmed to scan a 10 mm × 10 mm area 300 times in a rectangular pattern (the distance between lines was set to 40 μm). Laser scanning speed varied in the range of 2000–7000 mm/s. After the laser ablation process, the solvent color changed to light brown, indicating that Si NPs were dispersed in the styrene solution. For all prepared Si NP colloidal dispersions, the centrifugation (Lace16, COLO LabExperts, Buče, Slovenia) was performed at 10,000 rpm for 20 min in order to remove larger silicon particles.

Thin films containing Si NP were fabricated by dissolving (i.e., in a closed bottle heated at 80 °C for 8 h with periodic shaking of the mixture) 400 mg of ZEONEX^®^ in 4 mL of Si NP colloidal dispersion and drop casting a prepared mixture onto a glass coverslip (Menzel Gläser, Braunschweig, Germany) followed by evaporation of the styrene at 90 °C for 2 h. A free-standing film containing Si NP was obtained by gently peeling it from the glass coverslip. 

A transmission electron microscope (TEM) Tecnai G2 F20 X-TWIN was used to examine the as-prepared Si NP. The accelerating voltage was 200 kV. TEM images were processed with an interactive machine learning tool [53] for Si NP segmentation via the pixel classification method. Segmented Si NP images were analyzed using Fiji ImageJ (v.1.53t, Wayne Rasband and contributors, National Institutes of Health, Bethesda, MD, USA) distribution. The polydispersity index (PDI) was calculated from nanoparticle size distribution histograms using the following formula:

(1) $$PDI=\frac{\sqrt{\frac{\sum \left(\omega_{i}\cdot {\left(D_{i}-D_{avg}\right)}^{2}\right)}{\sum \omega_{i}}}}{D_{avg}}$$

where *ω_i_* is the weight fraction of nanoparticles in a particular size bin (in this case, the frequency in each size bin divided by the total frequency); *D_i_* is the diameter of particles in a particular size bin; *D_avg_* is the average diameter of the particles.

Raman spectra were recorded using an inVia Raman spectrometer (Renishaw, Wotton-under-Edge, UK) equipped with a CCD camera and confocal microscope (50× objective). The Raman spectra were excited with 532 nm radiation of a semiconductor green laser. The 2400 lines/mm grating was used to record the Raman spectra.

The photoluminescence (PL) spectra of the Si NP colloidal dispersion and thin film were recorded at room temperature with a luminescence spectrometer FLS980 (Edinburgh Instruments Ltd., Livingston, UK). PL decay curves of the Si NP colloidal dispersion and thin film were recorded at room temperature with the same spectrometer, using an LDH-D-C-375 laser (PicoQuant, Berlin, Germany) with a wavelength of 374 nm as the excitation source.

## 3. Results and Discussion

**Figure 1:**
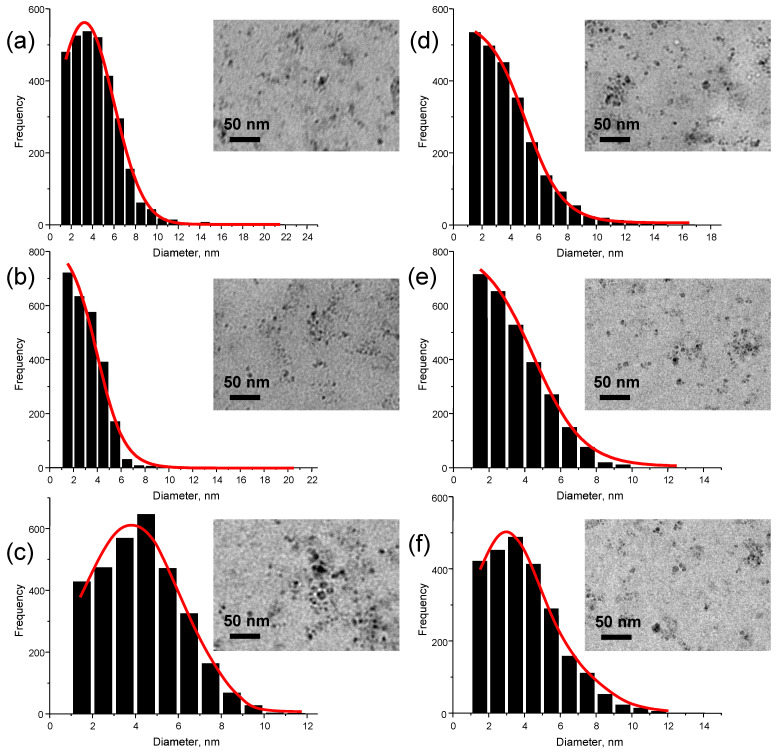
The size distribution of Si NPs produced using laser scanning speed of 2000 mm/s (**a**), 3000 mm/s (**b**), 4000 mm/s (**c**), 5000 mm/s (**d**), 6000 mm/s (**e**), and 7000 mm/s (**f**). Insets: typical TEM images of Si NP. Red lines correspond to the fitting.

By simply changing the laser scanning speed, the Si NP size distribution was altered considerably. Figure 1 shows the size distribution of Si NP produced at different laser scanning speeds, as well as the representative TEM images.

Through the analysis of TEM images, it was determined that most Si NPs are spherical. The PDI, an important parameter for evaluating nanoparticle size distribution, was found to be >0.7 in all instances, indicating a relatively wide distribution and heterogeneity in the formulations. The lowest PDI was found to be 0.811 for Si NP produced at 3000 mm/s laser scanning speed. In this case, the average nanoparticle diameter was found to be ~4 nm. The 3000 mm/s laser scanning speed was selected for further experiments as it was the most favorable choice to observe quantum confinement and size effects.

**Figure 2:**
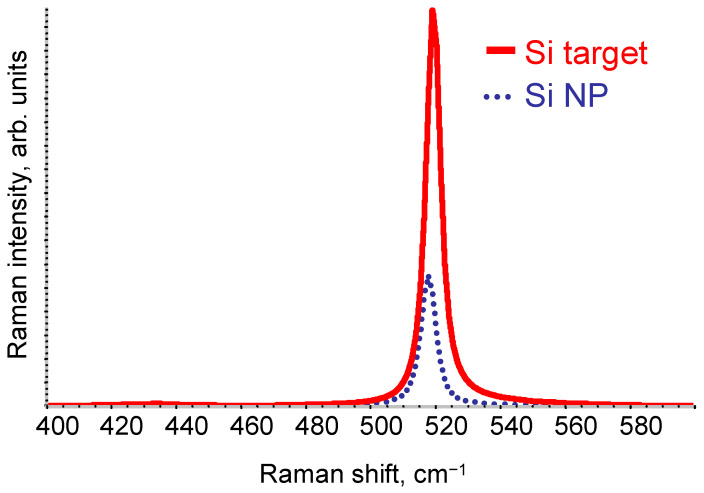
Raman spectra of the pristine Si target and Si NP (dotted line).

Figure 2 shows the Raman spectra of the pristine Si target and Si NP. In contrast to the pristine Si target, Si NP shows a hypsochromic shift of the Raman spectrum peak position (from 519.3 cm^−1^ to 517.8 cm^−1^) as well as a decrease in intensity. The latter could be due to the light scattering effects, while the hypsochromic shift may originate from the photon vibration confinement in the Si NP occurring due to the size effects [54,55]. This result is in good agreement with [41]. Tan Dezhi et al. produced Si NP via femtosecond laser ablation in 1-hexene under ambient conditions. They also observed a hypsochromic shift of the Si NP Raman spectrum peak position with respect to the pristine Si target.

**Figure 3:**
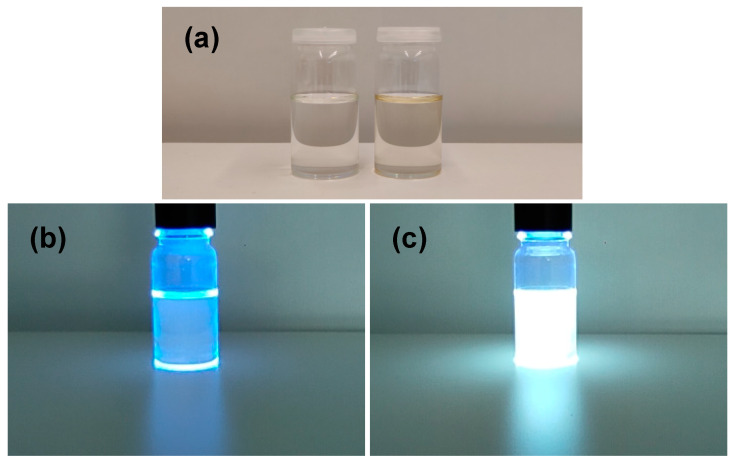
Digital photographs of the styrene solution (left) and Si NP colloidal dispersion (right) under ambient light (**a**). Digital photographs of the styrene solution (**b**) and Si NP colloidal dispersion (**c**) under photoexcitation at 365 nm.

Figure 3a shows digital photographs of the styrene solution and Si NP colloidal dispersion under ambient light and photoexcitation at 365 nm. It is important to note that Si NP colloidal dispersion remains stable for months in an ambient environment. Si NP colloidal dispersion under ambient light is transparent and light yellowish in color. Under photoexcitation at 365 nm, Si NPs exhibit what appears to be white color emission, which is clearly observable with the naked eyes in room light. Soojin Lee et al. developed a sonochemical route for the preparation of Si NP with a chlorine termination group [56]. These nanoparticles exhibited a spherical shape with a size ranging from 1 nm to 5 nm. Si NP also exhibited apparent white color emission under 360 nm photoexcitation. It was suggested that the white color emitting Si NP could be potentially applicable for optoelectronic devices (light emitting diodes, RGB displays, etc.).

**Figure 4:**
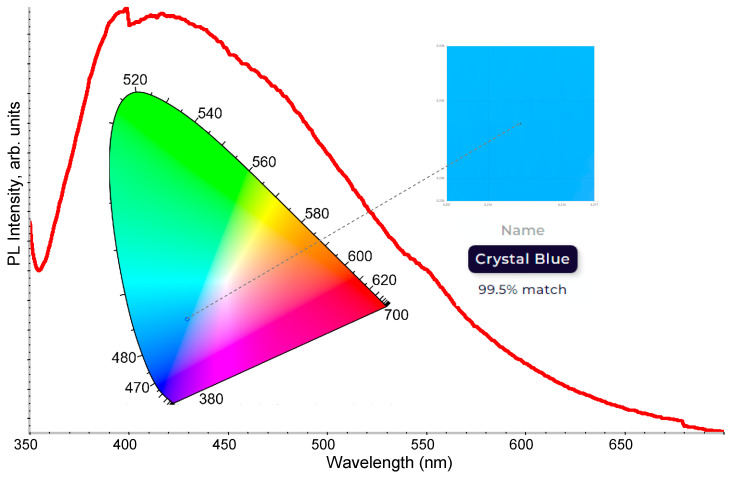
The characteristic PL emission spectrum of Si NP colloidal dispersion. The excitation wavelength of 330 nm was used. Inset: the CIE chromaticity diagram with emission color indication.

Figure 4 shows the characteristic PL spectrum of Si NP colloidal dispersion under an excitation of 330 nm. The broad spectrum covers a wide visible wavelength range with an emission peak centered at 416.5 nm. Please note that the anomaly observed in the spectrum around ~400 nm is due to the automatic filter replacement by the instrument during the PL measurement. The inset in Figure 4 depicts the Commission Internationale de L’Eclairage (CIE) chromaticity diagram of the Si NP colloidal dispersion with corresponding emission color determined from the PL spectrum. The Si NP colloidal dispersion exhibited a crystal blue color with color space coordinates of X: 1.0708, Y: 1.1785, and Z: 2.7970. 

**Figure 5:**
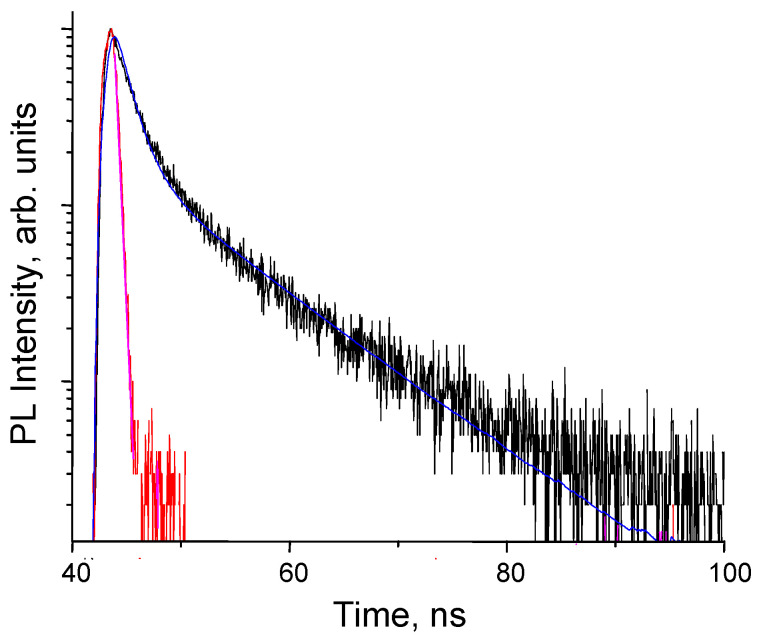
The PL decay curve of Si NP colloidal dispersion. Red and black lines represent experimental data, while blue and pink lines correspond to the bi-exponential fitting.

Figure 5 shows the PL decay curve of Si NP colloidal dispersion. Our analysis of the photoluminescence decay data revealed a biexponential decay behavior, as evidenced by the presence of two distinct lifetimes, τ_1_ = 1.38 ns and τ_2_ = 9.32 ns, respectively. The goodness of fit was evaluated using the reduced chi-squared value (χ^2^), which was found to be 1.068. This value indicates a reasonable fit to the experimental data. The biexponential photoluminescence decay behavior observed in Si NP implies the existence of multiple radiative recombination pathways within the nanoscale structure. The shorter lifetime (τ_1_) likely corresponds to a radiative recombination process involving defect states or surface-related emission. The longer lifetime (τ_2_) may originate from the intrinsic bulk-related recombination processes. In [41], the synthesized Si NP also exhibited the two photoluminescence lifetimes in the order of nanoseconds. Fast recombination rates of Si NP imply that electrons and holes recombine quickly, leading to efficient radiative recombination and light emission. In the context of optoelectronic devices like light-emitting diodes (LEDs) and lasers, this is highly desirable because it results in brighter and more intense emissions. Faster recombination also means that the device can respond to changes in electrical current or optical excitation more rapidly.

**Figure 6:**
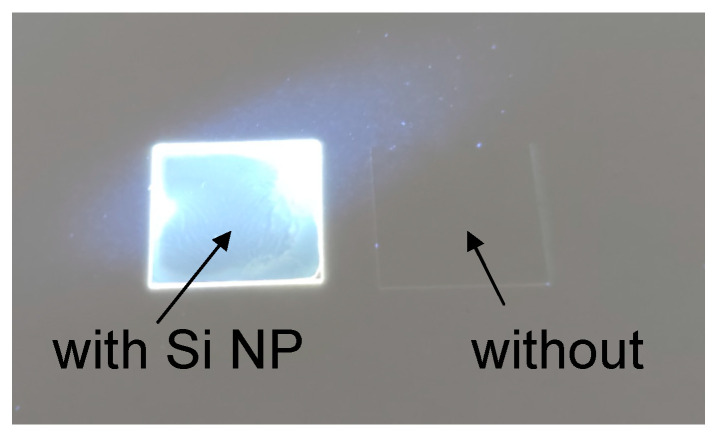
Digital photograph of the thin film containing Si NP (**left**) and without nanoparticles (**right**) under photoexcitation at 365 nm.

Figure 6 shows a photograph of the thin film containing Si NP and without nanoparticles under photoexcitation at 365 nm. Similarly, as for Si NP colloidal dispersion, a thin film containing Si NP exhibits an apparent white color emission under UV light. It is a good indication that the cyclo olefin polymer matrix had no dramatic effect on the photophysical properties of Si NP.

**Figure 7:**
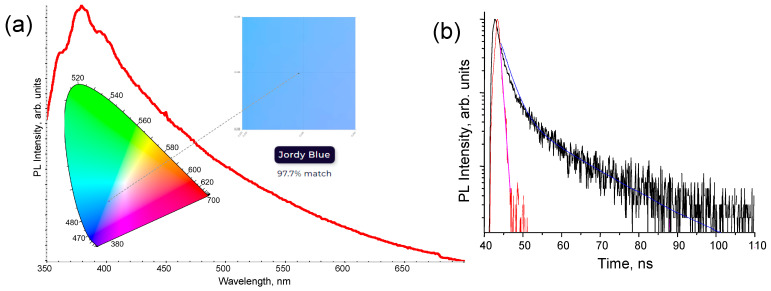
The characteristic PL emission spectrum of the free-standing thin film containing Si NP (**a**). The excitation wavelength of 330 nm was used. Inset: the CIE chromaticity diagram with emission color indication. PL decay curve of the free-standing thin film containing Si NP (**b**). Red and black lines represent experimental data, while blue and pink lines correspond to the bi-exponential fitting.

Figure 7a shows the characteristic PL spectrum of the free-standing thin film containing Si NP under excitation of 330 nm. In contrast to Si NP colloidal dispersion, the emission peak in the broad PL spectrum of the free-standing thin film containing Si NP was shifted to the lower wavelength from 416.5 nm to 379.2 nm. Additionally, the corresponding emission color was slightly changed from crystal blue to Jordy blue, with color space coordinates of X: 4.9239, Y: 5.0350, and Z: 1.1027 in the CIE chromaticity diagram. When Si NPs were embedded in the cyclo olefin polymer matrix, the size confinement of electrons and holes within the nanoparticles may have changed, affecting the energy bandgap and, consequently, the emission wavelength. Additionally, cyclo olefin polymer matrix can influence the exciton binding energy within the Si NP, which could also contribute to the shift in the emission energy and impact the recombination rate. Possible surface state changes of Si NP are also meaningful in this context [34]. For instance, surface passivation by oxygen and nitrogen leads to strong surface states, which may affect or even be decisive in governing the photophysical properties of Si NP [57,58,59]. In contrast to Si NP colloidal dispersion, the free-standing thin film containing Si NP exhibited biexponential decay (Figure 7b) with lifetimes of τ_1_ = 2.23 ns and τ_2_ = 13.75 ns, indicating a slower radiative recombination rate. The χ^2^ for the fit of the biexponential decay model to the experimental data was calculated to be 1.253, indicating a reasonable goodness of fit. The change in the Si NP environment and interactions with the cyclo olefin polymer matrix may have led to a reduction in non-radiative recombination processes [60]. Additionally, surface state changes, as mentioned earlier, could have influenced the number of surface traps or non-radiative recombination centers, potentially reducing their impact on PL decay. The overall photophysical changes were found to be acceptable as the PL spectrum of the thin film containing Si NP covered a broad visible wavelength range, and the change in radiative recombination rate was very small. 

**Figure 8:**
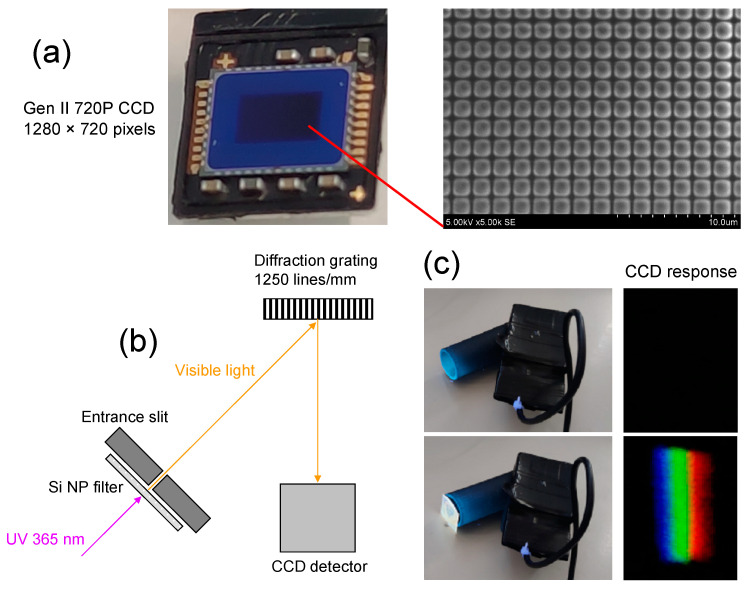
Digital photograph of the 2nd generation 720P CCD detector having a 1280 × 720 pixels active area (left) and scanning electron microscope micrograph of magnified active area with pixel matrix visible (right) (**a**). Simplified schematic of spectrometer configuration with a Si NP filter for UV light sensing via wavelength-shifting properties (**b**). Digital photographs of a working prototype in testing without the filter (top left) and with a Si NP filter (bottom left); the CCD detector response without the filter (top right) and with a Si NP filter (bottom right) upon exposure to 365 nm wavelength UV light (**c**).

In a continuous pursuit of identifying suitable nanoparticles for applications demanding the detection of UV light, the University of Texas at Arlington conducted experiments involving the preparation and testing of various luminescent nanoparticle samples [61]. Specifically, they tested a set of nanoparticles, such as ZnS:Mn [62], ZnS:Mn,Eu [63], CdTe [64], CuCy (copper cysteamine) [65], and LaF_3_:Ce [66], all of which are known for their capability to emit intense photoluminescence when exposed to UV excitations. These nanoparticles deposited directly on the surface of the photosensor improved its response over a large wavelength range of ~200–400 nm [61]. S. Magill et al. from the Argonne National Laboratory and the University of Illinois synthesized Si NP from Si wafers by chemical etching in hydrofluoric acid and hydrogen peroxide using electrical or hexachloroplatinic acid catalyst [67]. Si NPs were deposited on polymeric film and used as a passive filter for a standard visible-wavelength detecting photosensor. The response of the sensor was significantly enhanced at wavelengths <320 nm. In our experiments, a standard 2nd generation 720P CCD detector (Figure 8a) with a 1280 × 720 pixel active area (individual pixel size of ~1 μm) was used in a simple spectrometer configuration (Figure 8b), which utilized a fabricated Si NP film as a passive filter. This particular detector is not sensitive to UV light. As demonstrated in (Figure 8c), the CCD detector showed no response when exposed to 365 nm wavelength UV light during prototype testing (i.e., without the passive filter). In contrast, when the Si NP filter was employed in a similar test, the CCD detector exhibited red, green, and blue (RGB) color response, indicating that UV light was absorbed and re-emitted as white light. 

**Figure 9:**
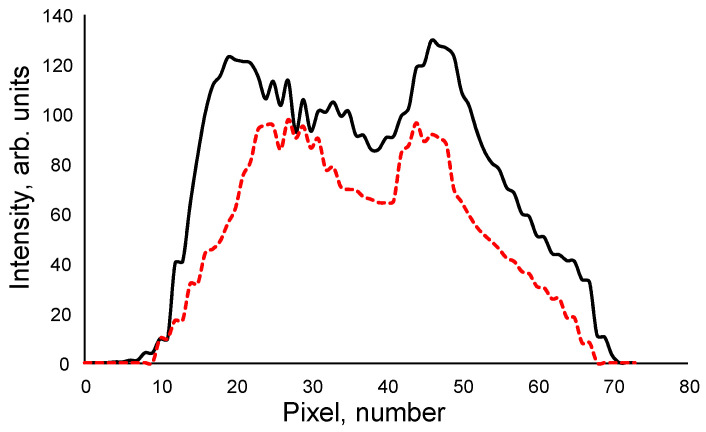
Typical response curves of the CCD detector when exposed to white light from the LED source (black line) and when exposed to UV light with a Si NP passive filter (red dashed line).

In a separate experiment, the CCD detector, without the filter, was exposed to white light from an LED source with an equivalent power rating to the UV light source, both at the same distance. The response of the CCD detector was recorded. Figure 9 shows the typical response curves of the CCD detector when exposed to white light from the LED source and when exposed to UV light with a Si NP passive filter.

Efficiency at each pixel was computed by taking the ratio of the intensity recorded with the filter to the intensity of the white light from the LED source. Weighted efficiency was determined by multiplying the efficiency at each pixel by the corresponding intensity of the white light from the LED source. The total weighted efficiency was then obtained by summing all the weighted efficiencies across all pixels. In a similar manner, the total intensity of the white light from the LED source across all pixels was calculated. Finally, the overall efficiency of the CCD detector with the Si NP filter was determined by dividing the total weighted efficiency by the total intensity of the white light from the LED source. The resulting overall efficiency of the CCD detector with the Si NP passive filter was determined to be 67 ± 2%. This result signifies the effectiveness of the Si NP passive filter in enhancing the CCD detector’s performance when exposed to UV light.

The following transformation enabled the non-sensitive CCD detector to detect UV light through the wavelength-shifting properties of Si NP. This example shows how a Si NP wavelength-shifting technology can be employed in a practical application to enhance the functionality of a CCD detector. Furthermore, it can be valuable for researchers and engineers working in fields related to optics, spectrometry, and sensor technologies. Beyond its immediate context, this practical example might inspire researchers to explore other applications of Si NPs in different areas of science and technology.

## 4. Conclusions

Silicon nanoparticles were produced using pulsed laser ablation in a styrene solution. Firstly, through precise control of the laser scanning speed during synthesis, we successfully modulated the size distribution of the Si NP. This parameter-driven size control allowed us to tailor the Si NP dimensions, with the optimal conditions identified at a scanning speed of 3000 mm/s, resulting in Si NP with an average diameter of ~4 nm and PDI of 0.811. The Raman spectra exhibited a hypsochromic shift in the peak position and a decrease in intensity compared to the pristine silicon target. Si NP colloidal dispersion excited at 330 nm unveiled a broad PL spectrum covering a wide visible wavelength range with a peak position at 416.5 nm. The Si NP colloidal dispersion exhibited a crystal blue color with color space coordinates of X: 1.0708, Y: 1.1785, and Z: 2.7970 in the CIE chromaticity diagram. PL decay analysis demonstrated a bi-exponential decay behavior, indicating the existence of multiple radiative recombination pathways within the nanoscale structure. Fabricated free-standing thin films containing Si NPs exhibited slightly different photophysical properties as compared to Si NP colloidal dispersions. The emission peak in the broad PL spectrum shifted to the lower wavelength from 416.5 nm to 379.2 nm, with the emission color change to Jordy blue. Additionally, free-standing thin films containing Si NP exhibited a slightly slower radiative recombination rate with lifetimes of τ_1_ = 2.23 ns and τ_2_ = 13.75 ns. Furthermore, we extended the practical use of Si NP by utilizing them as passive filters for CCD detectors. This practical example leveraged the wavelength-shifting properties of Si NP to enable non-sensitive CCD detectors to detect UV light. The overall efficiency of the CCD detector with the Si NP passive filter was determined to be 67 ± 2%. The transformation of UV light into visible light through Si NP-induced wavelength shifting showcased the versatility of Si NP in enhancing the functionality of existing technologies. These findings not only advance our fundamental understanding of Si NP but also pave the way for innovative applications in optoelectronics and related fields. As Si NPs continue to evolve as a key nanomaterial, their unique properties, including quantum confinement effects, hold promise for transformative advancements in diverse scientific and technological domains.

## Data Availability

Data are contained within the article.
